# Biological and physical approaches on the role of piplartine (piperlongumine) in cancer

**DOI:** 10.1038/s41598-020-78220-6

**Published:** 2020-12-17

**Authors:** Tiago Henrique, Caroline de F. Zanon, Ana P. Girol, Ana Carolina Buzzo Stefanini, Nayara S. de A. Contessoto, Nelson J. F. da Silveira, Daniel P. Bezerra, Edilberto R. Silveira, José M. Barbosa-Filho, Marinonio L. Cornélio, Sonia M. Oliani, Eloiza H. Tajara

**Affiliations:** 1 Department of Molecular Biology, School of Medicine of São José do Rio Preto (FAMERP), Av Brigadeiro Faria Lima 5416, São José do Rio Preto, SP CEP 15090-000 Brazil; 2 Department of Biology, São Paulo State University (UNESP), Institute of Biosciences, Humanities and Exact Sciences (IBILCE) - Campus São José do Rio Preto, Cristóvão Colombo, 2265, São José do Rio Preto, SP 15054-000 Brazil; 3 Integrated College Padre Albino Foundation (FIPA), Catanduva, SP 15806-310 Brazil; 4 Department of Physics, São Paulo State University (UNESP), Institute of Biosciences, Humanities and Exact Sciences (IBILCE) - Campus São José do Rio Preto, Cristóvão Colombo, 2265, São José do Rio Preto, SP 15054-000 Brazil; 5 Laboratory of Molecular Modeling and Computer Simulation/MolMod-CS, Institute of Chemistry, Federal University of Alfenas, Alfenas, MG 37130-001 Brazil; 6 Gonçalo Moniz Institute, Oswaldo Cruz Foundation (IGM-FIOCRUZ/BA), Salvador, BA 40296-710 Brazil; 7 Department of Chemistry, Federal University of Ceará, Fortaleza, CE 60020-181 Brazil; 8 Laboratory of Pharmaceutics Technology, Federal University of Paraiba, João Pessoa, PB 58051-900 Brazil; 9 Department of Genetics and Evolutive Biology, Institute of Biosciences, University of São Paulo, São Paulo, SP 05508-090 Brazil

**Keywords:** Biophysics, Cancer, Molecular biology, Structural biology

## Abstract

Chronic inflammation provides a favorable microenvironment for tumorigenesis, which opens opportunities for targeting cancer development and progression. Piplartine (PL) is a biologically active alkaloid from long peppers that exhibits anti-inflammatory and antitumor activity. In the present study, we investigated the physical and chemical interactions of PL with anti-inflammatory compounds and their effects on cell proliferation and migration and on the gene expression of inflammatory mediators. Molecular docking data and physicochemical analysis suggested that PL shows potential interactions with a peptide of annexin A1 (ANXA1), an endogenous anti-inflammatory mediator with therapeutic potential in cancer. Treatment of neoplastic cells with PL alone or with annexin A1 mimic peptide reduced cell proliferation and viability and modulated the expression of MCP-1 chemokine, IL-8 cytokine and genes involved in inflammatory processes. The results also suggested an inhibitory effect of PL on tubulin expression. In addition, PL apparently had no influence on cell migration and invasion at the concentration tested. Considering the role of inflammation in the context of promoting tumor initiation, the present study shows the potential of piplartine as a therapeutic immunomodulator for cancer prevention and progression.

## Introduction

Several epidemiological studies have indicated that persistent infection and chronic inflammation are predisposing factors for cancer, which is well-documented for the cervix after HPV infection^1^ and the stomach in the presence of *Helicobacter pylori*^2^. Supporting this idea are data from studies of tumor initiation and promotion, such as experiments using exogenous inducers of localized inflammation to promote cancer in mouse skin^3,4^ or xenotransplantation of colonic adenoma cells that only induce tumors if introduced into the host together with an inducer of inflammation^5^.

Mediators of inflammation, such as cytokines, chemokines, growth factors and free radicals, can provide a favorable microenvironment that fosters genome instability, survival, proliferation and migration and thus contribute to all cancer development stages, from initiation and promotion to metastasis^6^. This link between inflammation and tumorigenesis raises the possibility of therapeutic interventions that target inflammation for cancer prevention and treatment, especially because anti-inflammatory agents show a modest toxicity compared to conventional chemotherapy^7^.

An endogenous anti-inflammatory mediator with therapeutic potential in cancer is annexin A1 (ANXA1), a 37 kDa glucocorticoid–inducible protein that is involved in several biological processes, promoting apoptosis^8^, increasing migration and invasion^9,10^, and reducing^11^ or promoting^10^ cell proliferation and survival, a discrepancy that can be dependent on the cell type or differentiation stage^12^. Most of these biological effects of ANXA1, including regulation of inflammatory modulators, are mediated by its 26-amino-acid ANXA1 N-terminus peptide, Ac_2-26_, that is released from the protein through regulated proteolysis^13–16^.

Considering its involvement in these biological processes, it is possible to conclude that ANXA1 may directly participate in tumor initiation and progression^17,18^ and may be a tumor suppressor^19–21^. Abnormal expression is observed for their cognate partners, the formyl-peptide receptors (FPRs), a family of seven transmembrane G-protein-coupled receptors that, in addition to ANXA1 and its peptide, bind many proteins and peptides and may have opposite effects depending on the ligand^22,23^.

It has been shown that intracellular ANXA1 activates nuclear factor κB (NF-κB), which stimulates breast cancer cell invasion through increased expression of target genes^24^. Nuclear ANXA1 also appears to be involved in heavy metal-induced mutagenesis^25^ and is a predictor of decreased overall survival in oral squamous cell carcinoma^26^. Similarly, externalized ANXA1/FPR1 overexpression is associated with metastasis in gastric cancer^22,27^, tumor growth and invasion in gliomas^28^, and unfavorable prognostic factors in breast cancer^29^. Moreover, our group showed that nuclear and cytoplasmic ANXA1 and ANXA1/FPR2 are downregulated in dysplastic, primary tumor or metastatic laryngeal carcinoma^19,20,30^, suggesting that the role of ANXA1 in tumorigenesis is context-dependent.

The anti-inflammatory role of ANXA1 is partially due to its ability to inhibit cytosolic phospholipase A_2_ (cPLA_2_) activity and, consequently, the hydrolysis of membrane phospholipids and the release of arachidonic acid. Overexpression of cPLA_2_ and induction of arachidonic acid metabolization result in high levels of eicosanoids, such as prostaglandin E_2_, which are frequently observed in tumors^31^. These data support the use of aspirin and other nonsteroidal anti-inflammatory drugs for cancer prevention, which, although effective, are associated with several adverse side effects^32^. It is therefore important to search for new medications that control inflammation and show a more acceptable safety profile.

Piplartine (PL), investigated with X-ray diffraction by Boll et al.^33^ and named piperlongumine [5,6-dihydro-1-[(2E)-1-oxo-3-(3,4,5-trimethoxyphenyl)-2-propenyl]-2(1H)-pyridinone), PubChem CID 637858; Zinc00899053], is a plant-derived small molecule, which has been the subject of reports from members of our group^34–37^. PL is a biologically active alkaloid/amide from long pepper (*Piper longum*) with many reported pharmacological properties, including anti-inflammatory and antitumor activity by induction of oxidative stress and low toxicity^38,39^. The presence of a trimethoxy aromatic ring in the PL structure may favor its interaction with tubulin^40^ and its role as a microtubule-destabilizing agent with anti-proliferative effects^41^. Similar to annexin A1, PL is involved in NF-κB and MAPK signaling pathways^42–44^.

In the present study, we investigated physical and chemical evidence of interaction between PL and an annexin A1-derived peptide Ac_2-26_ and its potential effects on cell proliferation, viability, migration, apoptosis, inflammatory responses, and gene expression of inflammatory mediators.

## Results

The present study investigated the action of PL on two cell lines derived from normal or neoplastic tissues, HUVEC and HEp-2, respectively, as well as the potential effect of PL and PL-ANXA1 peptide Ac_2-26_ interaction on biological processes related to inflammation and cancer.

### Molecular docking

Molecular docking tool was employed using 14 human proteins related to inflammatory and neoplastic processes as targets, and PL and eight anti-inflammatory compounds as ligands (Supplementary Table S1). The data showed that PL exhibits binding free energy values similar to those of anti-inflammatory compounds (Supplementary Table S2), and also potential interaction with the N-terminal of ANXA1 corresponding to the Ac_2-26_ peptide (Supplementary Fig. S1).

**Figure 1:**
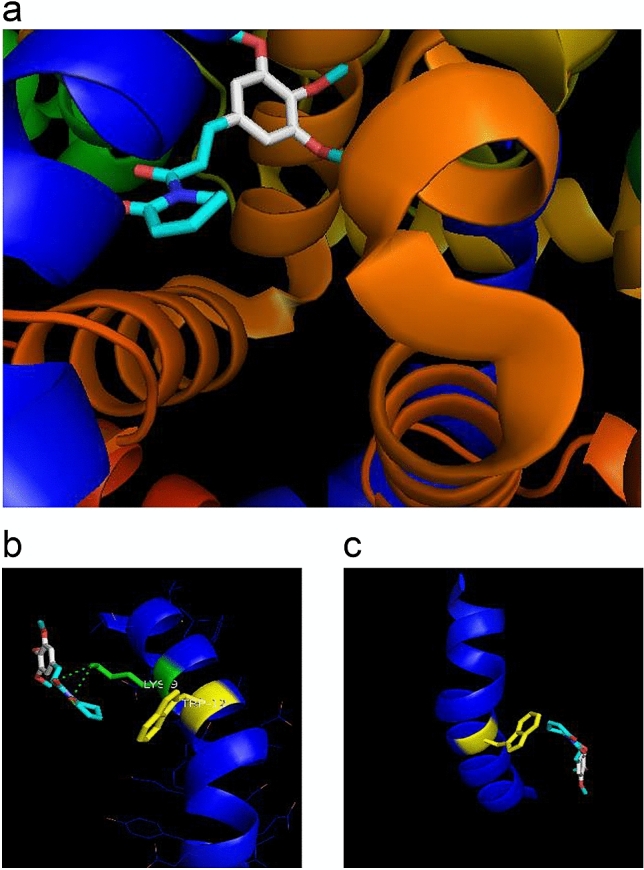
The docked position of PL onto the Ac_2-26_ peptide. (**a**) Secondary structure of annexin A1 is represented in orange, Ac_2-26_ peptide in dark blue and PL as sticks. (**b**, **c**) As shown, the Ac_2-26_ peptide binds to PL via two hydrogen bonding interactions (dotted lines) at lysine 9 (in green) but not at tryptophan 12 (in yellow). Data were taken, with permission, from experiments performed by the author Henrique T^95^, and figure printed in^96^. Figure was generated by Pymol system version 2.0 (https://pymol.org/).

The docking method was validated by redocking the co-crystallized ligands to MAPK 1, MAPK 14 and TNF-a (MAP kinase 1, MAP kinase 14 and tumor necrosis factor, respectively). The results showed that the redocked ligand positions are similar to the crystallized positions, with a positional root mean square deviation (RMSD) below the tolerance level; therefore, the protocol was reliable to predict the binding conformation of ligands. The potential interactions obtained by the docking position of PL with Annexin A1 are located in the sequence that corresponds to the Ac_2-26_ peptide. Moreover, the Ac_2-26_ peptide binds to piplartine via two hydrogen bonding interactions at lysine 9 (Fig. 1). Five anti-inflammatory compounds in current clinical use (acetaminophen, ketorolac, naproxen, nimesulide, and resveratrol) also showed potential interactions with the N-terminal sequence of Annexin A1 corresponding to Ac_2-26_ peptide (Supplementary Fig. S1). PL and Ac_2-26_ peptide were thus selected for subsequent in vitro experiments.

### UV–Vis absorbance and fluorescence spectroscopy

**Figure 2:**
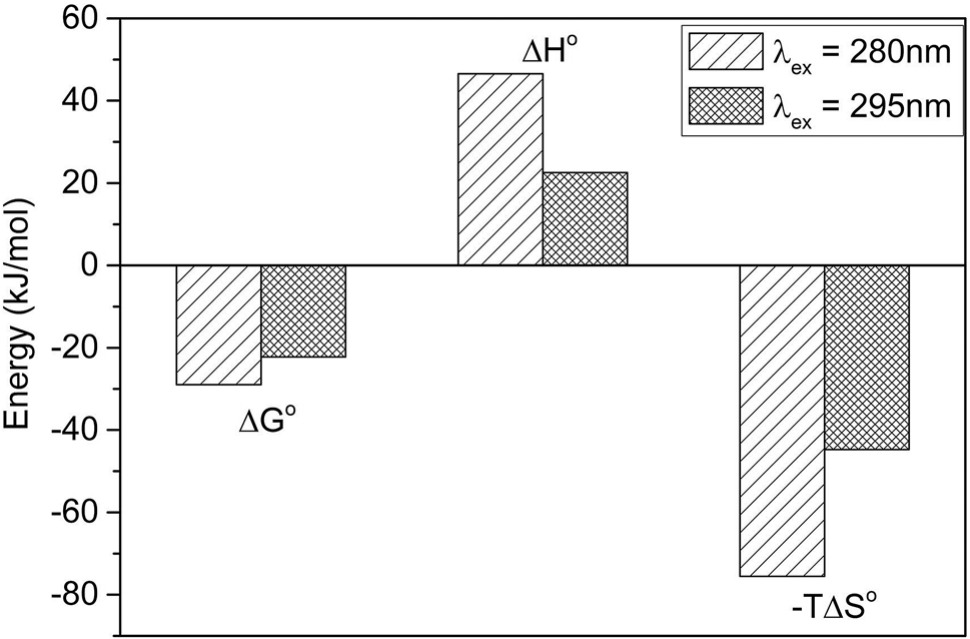
Energy contributions to the interaction between Ac_2-26_ and PL at different excitation wavelengths (280 nm and 295 nm). Monitoring the microenvironment of the interaction around the aromatic tryptophan residue. ΔG = Gibbs free energy changes, ΔH = enthalpy changes, ΔS = entropy changes, T = temperature. Data were taken, with permission, from experiments performed by the authors Contessoto NSA and Cornélio ML, and figure printed in^96^.

Fluorescence emission data (Supplementary Fig. S2) were plotted in a double logarithmic frame (Supplementary Fig. S3) to obtain energy values involved in the Ac_2-26_ peptide and piplartine interaction^45^, and the van't Hoff analysis was performed to determine the thermodynamic parameters. According to the sequence of Ac_2-26_, the presence of three endogenous fluorescence probes enhanced the probability of checking distinct regions of the peptide undergoing interaction. Two phenylalanine (F) probes at positions 6 and 12 and one tryptophan (W) at position 11 favorably expanded the search in two sectors of the peptide. Free energy changes were monitored by the changes in emission spectra during the titration. Excitation wavelengths of 280 nm and 295 nm excited F and W probes, respectively. At both excitation wavelengths, the enthalpy and entropy variations were positive (ΔH > 0 and ΔS > 0), and the Gibbs free energy variation was negative (ΔG < 0), as shown in Fig. 2, suggesting that the interaction of PL with the annexin A1-derived peptide Ac_2-26_ occurs spontaneously (Supplementary Table S3). For more details, see Supplementary Information and references^46–48^.

### PL and Ac_2-26_ modulate proliferation and viability

**Figure 3:**
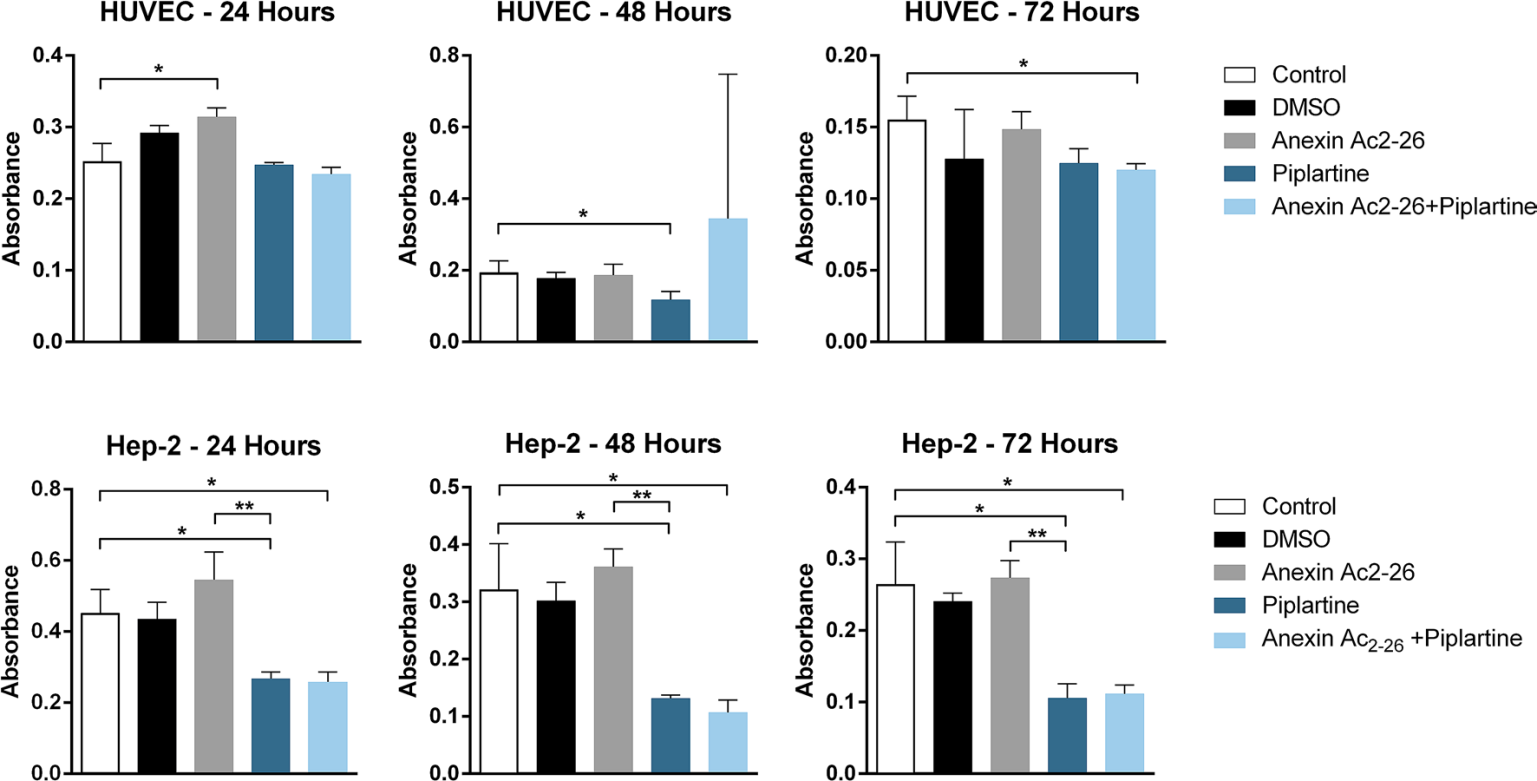
PL and Ac_2-26_ treatments decrease the number of viable HEP-2 cells, but have a low effect on HUVEC viability. MTS assay was used to determine proliferation and viability of neoplastic and normal cells treated with PL or Ac_2-26_ alone or in combination for 24 h, 48 h and 72 h. An equivalent volume of vehicle (final concentration in culture medium = 10 µg/mL) without PL or Ac_2-26_ was added to the DMSO control group, and no DMSO/PL/Ac_2-26_ to the negative control. Assays were carried out in triplicate, and experiments were performed two times (ANOVA *p < 0.05; **p < 0.001).

To investigate whether Ac_2-26_ and/or PL influence normal and carcinoma cell survival, HUVEC and HEp-2 cells were treated with PL and/or Ac_2-26_, and MTS assays were read at 24, 48 and 72 h. Treatment of HUVEC cells with Ac_2-26_ displayed a low effect on cell proliferation and viability. Otherwise, a significant decrease in the number of viable HEp-2 cells was observed after incubation with PL (Fig. 3).

### PL effect on cell migration and invasion

**Figure 4:**
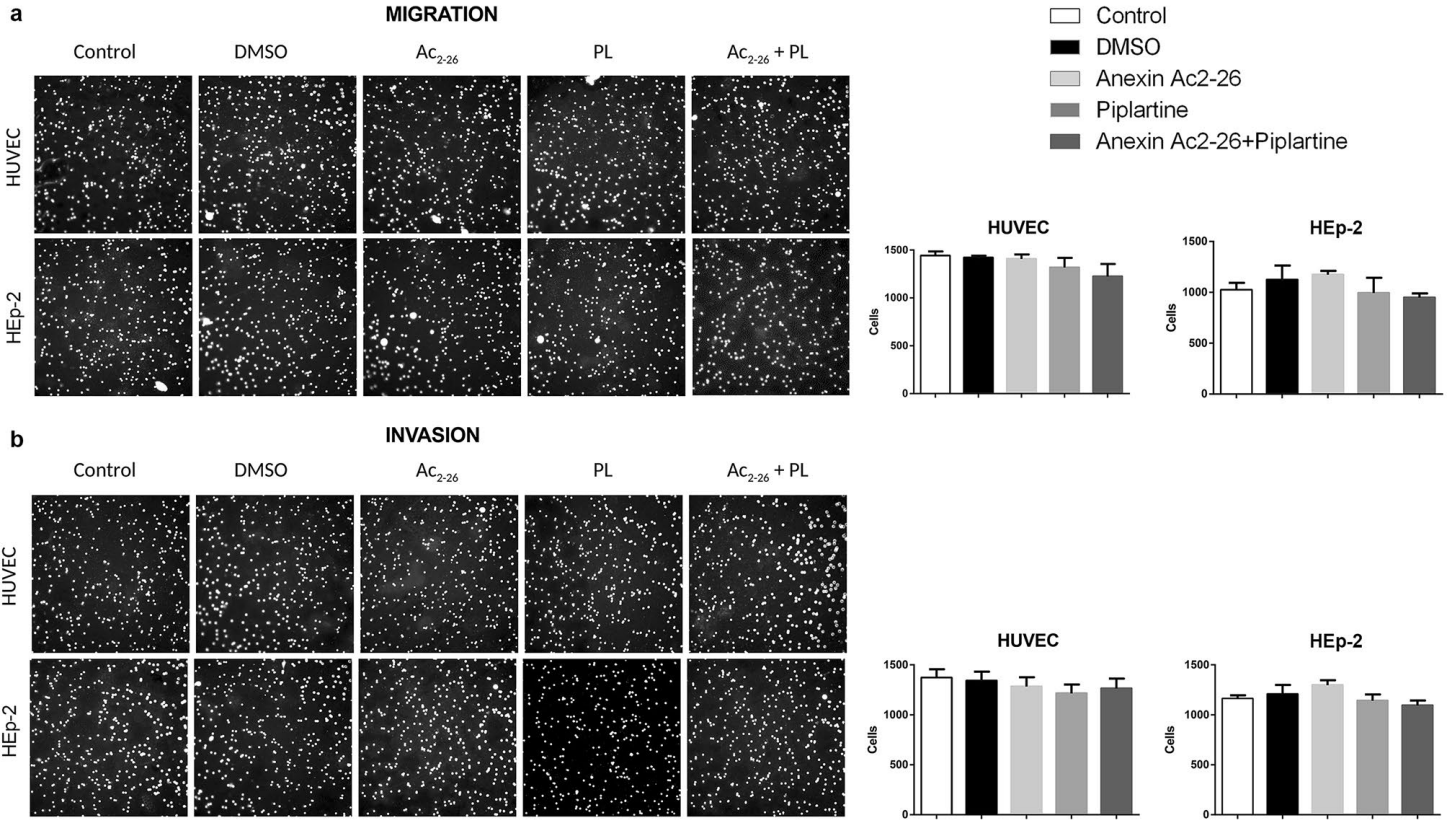
PL apparently has no effect on migration and invasion of HUVEC and HEp-2 cells. (**a**) Representative images of cell migration and (**b**) invasion assays performed on cells treated with PL alone or in combination with Ac_2-26_ (cells stained by DAPI) (left panels). Quantitative analysis of the number of migrated and invasive cells relative to untreated controls (right panels). An equivalent volume of vehicle (final concentration in culture medium = 10 µg/mL) without PL or Ac_2-26_ was added to the DMSO control group, and no DMSO/PL/Ac_2-26_ was added to the negative control. Assays were carried out in triplicate (ANOVA *p < 0.05; **p < 0.001).

To investigate whether PL could affect metastasis of carcinoma cells, the effects of PL on cell migration and invasion were analyzed. As shown in Fig. 4, the PL alone or in combination with Ac_2-26_ treatment for 24 h and 48 h apparently had no effect on migration and invasion, respectively, of normal HUVEC and HEp-2 cells.

### PL inhibits α-tubulin expression

**Figure 5:**
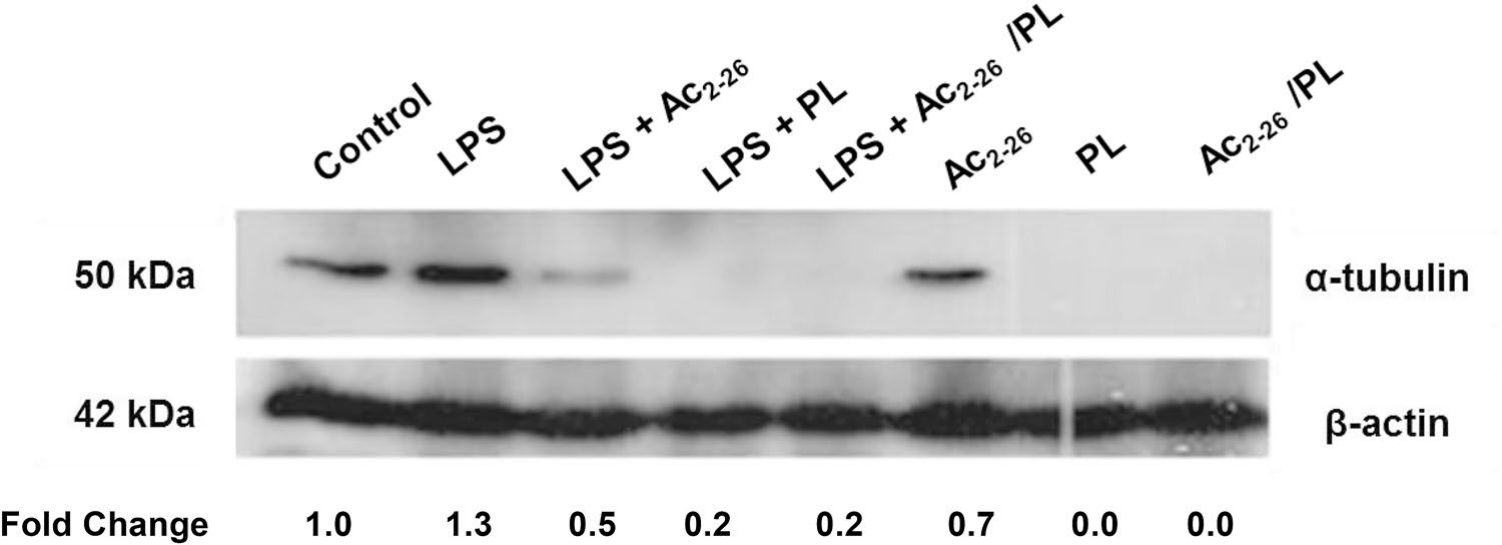
PL inhibits α-tubulin expression. Western blotting analysis of HUVECs treated with PL alone or in combination with Ac_2-26_ and/or LPS shows that PL inhibits α-tubulin (50 kDa) expression. β-actin (42 kDa) was used as an endogenous control. No LPS/PL/Ac_2-26_ was added to the negative control. Fold change relative to control was calculated using the α-tubulin: β-actin ratio. Reproduced with permission from^96^. The vertical streaks on the blot images were probably caused by a contaminant on the scanning lamp/lens assembly glass.

Western blotting data demonstrated the absence of α-tubulin in HUVECs treated with piplartine alone or in combination with Ac_2-26_ and/or LPS, suggesting that PL directly or indirectly affects cytoskeleton reorganization. As expected, the endogenous control showed similar immunoreactivity in all experimental conditions (Fig. 5 and Supplementary Fig. S4).

### PL modulates chemokine and cytokine expression

**Figure 6:**
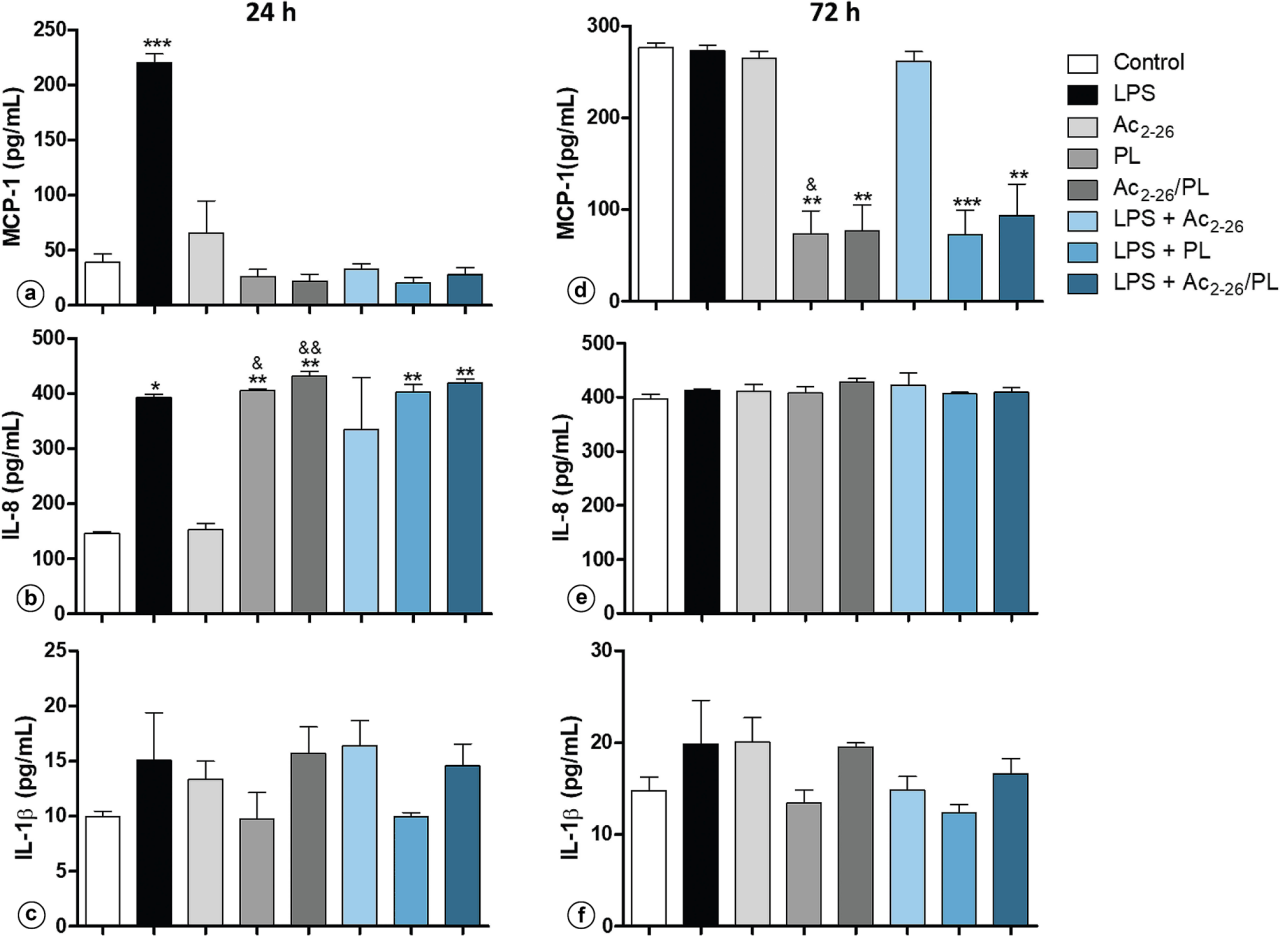
PL modulates chemokine and cytokine expression. ELISA analysis of proinflammatory cytokines MCP-1 (**a**, **d**), IL-8 (**b**, **e**), and IL-1β (**c**, **f**) from culture supernatants of HUVECs treated with LPS, Ac_2-26_ and/or PL for 24 and 72 h. No LPS/PL/Ac_2-26_ was added to the negative control. Results represent the mean ± SEM from of three independent assays (n = 3). *p < 0.05, **p < 0.01, and ***p < 0.001 versus untreated control; ^&^*p* < 0.05, ^&&^*p* < 0.05 versus Ac_2-26_. Reproduced with permission from^96^.

The effects of PL, Ac_2-26_ and LPS on chemokine and cytokine expression were investigated using HUVECs. The cells were grown under different experimental conditions, and the supernatants were collected for the determination of the expression of MCP-1 chemokine and IL-8 and IL-1β cytokines after 24 and 72 h. At the 24 h time point, a significant increase in the levels of the proinflammatory MCP-1 chemokine (Fig. 6a) was observed in the LPS-treated cells, and only LPS and PL group increased IL-8 levels after 24 h (Fig. 6b). The Ac_2-26_ peptide did not significantly alter the MCP-1 and IL-8 levels compared to the controls. No significant differences between groups in relation to IL-1β levels were detected (Fig. 6c).

At the 72-h time point, the levels of MCP-1 and IL-8 were high in the controls and no effect of LPS on modulating cytokines was observed (Fig. 6d,e). Treatment with PL alone or in combination with LPS and/or Ac_2-26_ induced a reduction of the MCP-1 concentration, whereas the Ac_2-26_ peptide alone showed no effect (Fig. 6d). Regarding IL-8 and IL-1β levels, no significant differences were observed in any of the groups studied (Fig. 6e,f).

### PL modulates the expression of genes involved in inflammatory processes

To explore the role of PL in inflammatory processes, expression of 92 genes related to the inflammatory response were quantified in PL-treated carcinoma cells and compared to untreated controls. A gene ontology analysis using DAVID tools^49,50^ was performed. As expected for a PCR array targeting genes involved in inflammatory processes, the highest ranked molecular functions for differentially expressed genes after PL treatment were phospholipase C, leukotriene receptor, phosphatidylinositol phospholipase C, receptor signaling protein, MAP kinase, G-protein coupled peptide receptor and signal transducer activities (Benjamini–Hochberg method, *p* values < 0.05, Supplementary Table S4). Twenty-five genes showed decreased expression in treated cells compared to untreated cells; in particular, the genes encoding phospholipases, leukotrienes and interleukin receptors, serine/threonine kinases, and TNF-activated receptor. The 18 genes with increased expression in PL-treated cells included those encoding phospholipase A2, cytokine and interleukin receptors, endopeptidases and the transcriptional regulator TNF (Table 1). Two major canonical pathways identified by the INGENUITY PATHWAY ANALYSIS (IPA) for the set of differentially expressed genes were Vitamin C Antioxidant Action and Eicosanoid Signaling (*p* values 5.27E^−15^, 6.10E^−15^, respectively). The five upstream regulators of these pathways with low *p* values were TNF, IL1B, IL4, LPS and CD40 (*p* values of overlap = 6.09E^−15^ to 1.20E^−10^), and the major diseases and biological functions were Cardiovascular Disease, Response Inflammatory Disease, Immune Disease, Inflammatory Disease and Respiratory Disease (*p* values = 7.43E^−20^ to 1.21E^−05^)^51^.

**Table 1:** PL modulates expression of genes involved in inflammatory processes.

Gene symbol	Molecular function^a^	Fold change	Gene symbol	Molecular function^a^	Fold change
*LTB4R2*	Leukotriene receptor	− 8.13	*TNF*	Transcription regulator	7.89
*LTB4R*	Leukotriene receptor	− 2.21	*IL2RA*	Interleukin-2 receptor	6.00
*PLCE1*	Phospholipase C	− 2.15	*IL1R2*	Interleukin-1 receptor	5.90
*PDE4D*	Phosphodiesterase	− 2.13	*PLA2G2A*	Phospholipase A2	3.90
*TBXA2R*	Thromboxane A2 receptor	− 2.12	*ITGAM*	Glycoprotein binding	3.91
*TNFRSF1A*	TNF-activated receptor	− 2.11	*CYSLTR1*	Cys-leukotriene receptor	2.98
*PTGER2*	Prostaglandin receptor	− 2.06	*HTR3A*	Serotonin receptor	2.97
*IL2RB*	Interleukin-2 receptor	− 2.06	*ALOX12*	Arachidonate 12-lipoxygenase	2.94
*ITGB1*	Fibronectin binding	− 1.17	*ADRB2*	Beta2-adrenergic receptor	2.91
*MAPK8*	Serine/threonine kinase	− 1.16	*CD40*	Signal transducer	2.87
*MAPK14*	MAP kinase	− 1.15	*IL1RL1*	Cytokine receptor	2.87
*MAPK1*	Serine/threonine kinase	− 1.15	*KLK2*	Endopeptidase	2.87
*BDKRB1*	Bradykinin receptor	− 1.13	*KLK3*	Endopeptidase	2.87
*HRH1*	Histamine receptor	− 1.12	*KNG1*	Endopeptidase inhibitor	2.87
*CACNB4*	Calcium channel	− 1.11	*PLA2G5*	Phospholipase A2	2.87
*PLCB3*	Phospholipase C	− 1.11	*NOS2*	Nitric-oxide synthase	2.72
*LTA4H*	Leukotriene-A4 hydrolase	− 1.11	*CES1*	Hydrolase	1.91
*A2M*	Interleukin-1/TNF binding	− 1.10	*KLK14*	Endopeptidase	1.88
*PLCG2*	Phospholipase C	− 1.10			
*NFKB1*	Transcription factor binding	− 1.09			
*NR3C1*	Transcription factor	− 1.09			
*PDE4B*	Phosphodiesterase	− 1.07			
*PLCG1*	Phospholipase C	− 1.06			
*ADRB1*	Receptor signaling protein	− 1.05			
*CACNB2*	Calcium channel	− 1.04			

Differentially expressed [log2 (fold change]) genes identified by a PCR array (TaqMan Array Human Inflammation 96-well plate) in HEp-2 cells treated with PL compared to untreated cells. Genes with log2 fold change > 1.0 were considered as differentially expressed. Reproduced with permission from^95^.

^a^According to Gene Ontology Consortium (http://geneontology.org/)^49,50^.

## Discussion

In the present study, the role of piplartine, a natural substance extracted from the *Piper longum* pepper, was evaluated for its activity as a ligand of the endogenous protein annexin A1 mimic peptide by favoring or attenuating its anti-inflammatory effects. PL was also analyzed in vitro for its biological functions linked to inflammation and tumor development.

Docking analysis of PL with annexin A1 showed that potential interactions between these compounds are located in the sequence corresponding to the Ac_2-26_ peptide; therefore, the peptide was used in functional experiments together with PL.

Computational and spectroscopy tools used in conjunction with each other were essential to evaluate physical aspects of the Ac_2-26_-PL interaction. The peptide presents endogenous fluorophores, which helped titration experiments. The responses of P6, P12 and W11 residues were very similar, though at different positions of the peptide. The thermodynamic parameters confirmed a favorable interaction between Ac_2-26_ and PL due to negative Gibbs free energy calculations. Our results suggested that the molecular structures and charge distribution in both compounds were appropriate for stereochemical recognition, which was sustained by considering the role of enthalpy and entropy contributions. The data also showed that the energy balance was dictated by entropy rather than enthalpy. No covalent bonds were formed, and the complex was likely stabilized by weak interactions, indicating that electrostatic interactions may have a key role in the formation of the complex.

In vitro analyses were conducted to explore whether piplartine and/or Ac_2-26_ alters proliferation and viability of cells derived from normal or neoplastic tissues and, consequently, affects inflammatory responses. The results showed that Ac_2-26_ has a low effect on HUVEC proliferation and viability.

Previous studies from our group^52^ have already shown that the peptide induces cell proliferation in HUVECs, both under basal conditions and after stimulation by VEGF. Other studies using inflammation and tumor models evidenced that ANXA1 may stimulate proliferation and migration^53^ or even inhibit proliferation^54^, most likely depending on the context of the experiment, cell type or tissue. Treatment of HUVECs with PL, alone or together with Ac_2-26_, also showed a low effect on cell viability. Differently, treatment of HEp-2 with PL significantly reduced the number of viable cells and counteracted the proliferative effect of annexin. Similarly, Chen et al.^55^ using other cell lines, both from oral squamous cell carcinomas (OCSL and OC2), found that PL inhibits cell growth and induces apoptosis.

PL appeared to have no effect on the migration and invasion ability of normal or neoplastic cells, which may be a consequence of the PL concentration used in the experiments. Really, similar findings have been observed by some authors in cell lines treated with low PL concentrations and, on the contrary, an evident reduction in migration and invasion with concentrations higher than 10 μM^56–59^.

Previous studies from our group had already showed that PL is able to reduce leukemic cell survival regulating cell death by caspase-dependent apoptosis and/or necrosis, an effect that appears to be selective for tumor cells^35^ and not related to cell membrane damage^36^. Bezerra and collaborators also observed that PL increases the antitumor activity of chemotherapeutic drugs in both in vitro and in vivo experimental models^37^ and induces G2/M cell cycle arrest, most likely due to its genotoxicity^34^. In addition, the authors commented that the compound is cytotoxic to tumor cell lines and that this may be due to the presence of two α- and β-unsaturated carbonyl radicals^36^. Other groups have expanded the knowledge of the biological properties of PL and suggested, inter alia, that PL inhibits hypoxia inducible factor-2 (HIF-2) transcription^60^ and modulates redox and ROS homeostasis^61,62^.

The presence of a trimethoxy aromatic ring in the PL structure, which may favor an interaction with tubulin^40^, and the fact that PL may be considered a microtubule-destabilizing agent with antiproliferative effects^41^ provide evidence that PL affects tubulin polymerization. Microtubules comprise protofilaments containing repeating α/β-tubulin heterodimers and contribute to cell shape and chromosome segregation. During the course of the cell cycle, interphasic microtubules generate mitotic and meiotic spindles, which allows the correct distribution of the chromosomes at cell division^63^. Microtubule dysfunction may result in chromosomal instability, mitotic arrest and cell death^64^. The results of the present study, in addition to the antiproliferative and antiviability effects of PL, showed that the treatment of a normal cell line with PL, alone or together with LPS or Ac_2-26_, inhibited the expression of α-tubulin.

The results obtained in the present study also showed that LPS increased pro-inflammatory MCP-1 chemokine and IL-8 cytokine expression in endothelium-derived cells, an expected result considering the properties of LPS^65,66^. Piplartine, alone or in combination with LPS and/or Ac_2-26_, also induced high levels of IL-8 but reduced the MCP-1 levels, with no effect on annexin performance. A recent study showed that high IL-8 levels may result in low capillary activity not related to cell viability^67^. IL-8 is a member of the CXC chemokine family that regulates endothelial cell migration, proliferation and angiogenesis^68^, and high levels of IL-8 reduce capillarization in vitro and in ex vivo systems^67^. Thus, we can conclude that piplartine effects on cell survival may be in part due to PL-mediated IL-8 expression.

To better understand the role of PL in modulating inflammatory mediators associated with tumorigenesis, the expression of 92 genes was analyzed in carcinoma cells. Among the genes that showed altered expression after PL treatment are receptors and enzymes linked to leukotriene biosynthesis (*LTA4H*, *LTB4R*, *LTB4R2*), prostaglandin (*PTGER2*), interleukin (*IL2RB*) and adrenergic (ADRB1) receptors, G-protein coupled receptors (*BDKRB1*, *HRH1*, *TBXA2R*), membrane receptors involved in cell adhesion (*ITGB1*), regulators of inflammatory response (*TNFRSF1A*), members of signaling pathways (*A2M*, *MAPK1*, *MAPK8*, *MAPK14*, *PDE4B*, *PDE4D*, *PLCB3*, *PLCE1*, *PLCG1*, *PLCG2*), transcriptional regulators (*NFKB1*, *NR3C1*) and calcium channel subunits (*CACNB2*, *CACNB4*). The genes of two phospholipases (*PLA2G2A* and *PLA2G5*), tumor necrosis factor (*TNF*), interleukin and cytokine receptors (*CD40*, *IL1RL1*, *IL2RA* and *IL1R2*) and leukotriene (*CYSLTR1*) had high expression after treatment (biological processes obtained from^69,70^).

The altered expression of these genes confirmed the action of piplartine as a substance capable of regulating the synthesis or activity of important members of the inflammatory cascade. Leukotrienes (LTs) and prostaglandins (PGs) are two good examples. These compounds are representatives of the class of eicosanoid lipids derived from the arachidonic acid released from the membrane by phospholipase A2 and are subsequently oxygenated by the lipoxygenase (LOX) and cyclooxygenase (COX) pathways, respectively^71^. Both leukotrienes and prostaglandins act on homeostasis and inflammation through G protein-coupled receptors and can be blocked by nonsteroidal anti-inflammatory drugs^72^. Prostaglandins are synthesized by most cells and act in autocrine and paracrine manners, whereas leukotrienes, both cysteine (cys-LTs) and LTB4, are generated by inflammatory cells after stimulation triggered by exogenous factors or events of intracellular phosphorylation^73,74^.

Proinflammatory signaling initiated by leukotriene LTB4 through its BLT1 and BLT2 receptors (encoded by *LTB4R* and *LTB4R2* genes) is associated with various diseases, such as asthma, rheumatoid arthritis, atherosclerosis, abdominal aortic aneurysm, multiple sclerosis, and cancer. Signaling by cysteinyl leukotriene (Cys-LT) receptors, in turn, has emerged as a key component of vascular inflammation, with an important role in the pathogenesis and progression of cardiovascular diseases^75^. Prostaglandin PGE2 and its cognate receptors (EP1-4, encoded by *PTGER1-4*) are also involved in many processes, including vascular permeability, cell proliferation and cell migration^71^. In vitro studies have shown that the signaling pathway triggered by prostaglandins and the EP2 receptor in the presence of lipopolysaccharides is associated with both anti-inflammatory effects and proinflammatory effects and that these responses depend on the type of stimulus and on the type of immune cell in which they were activated^76^.

Prostaglandins have been extensively studied, and high levels of PGE2 have already been observed in several subtypes of cancer. As a mediator of inflammation, PEG2 is involved in tumor growth and progression and, together with LTB4, can stimulate signaling pathways involving phospholipases (PLCs), phosphodiesterases (PDEs), cyclic nucleotides, inositol triphosphate (IP3), calcium channels, phosphatidylinositol 3 (PI3K) and MAPK kinases, resulting in different events, such as proliferation, angiogenesis, migration, invasion and survival^77,78^. Several of these effectors showed altered gene expression in HEp-2 cells after piplartine treatment.

Proinflammatory proteins, such as those acting as interleukin receptors and linkers (encoded by the *A2M*, *IL2RB* and *TNFRSF1A* genes), transcription factors (encoded by the *NFKB1* and *NR3C1* genes), phospholipases C, were also inhibited by PL; all of these proteins are known promoters of inflammation. Phospholipase C catalyzes the hydrolysis of phosphatidylinositol 4,5-bisphosphate in two secondary messengers, diacylglycerol and inositol 1,4,5-triphosphate. Cui et al.^79^ observed that the elevation of *PLCE1* (phospholipase C epsilon 1) expression in patients with esophageal carcinoma is associated with lymph node metastasis and staging of the lesion. The silencing of this enzyme in bladder carcinoma cells led to a reduction in the levels of metalloproteinases and the Bcl-2 apoptosis regulator, consequently decreasing invasion activities^80^.

Most results on gene expression analysis support the anti-inflammatory and antitumoral properties of piplartine. The explanation of PL-mediated induction of proinflammatory genes may lie on the simultaneous dual pro- and anti-inflammatory activity of some proteins or on negative feedback mechanisms. This dual activity depends on the cellular context and is observed for several inflammatory cytokines, such as TNF (tumor necrosis factor). TNF plays a central role in the pathogenesis of some inflammatory diseases and has been extensively studied by mediating important biological processes, including cell proliferation, survival, and death^42,44^. Deregulation of these processes is a characteristic of inflammation and cancer. The signal transduction pathway of TNF is complex. Responses to this factor are triggered by the activation of one of its receptors, TNFR1 and TNFR2 (encoded by the *TNFRSF1A* and *TNFRSF1B* genes, respectively). The extracellular domains of these receptors are homologous and have similar affinities for TNF, but the cytoplasmic regions are distinct and activate signaling events with different biological effects. While TNFR1 contains the death domain, TNFR2 does not. Thus, depending on the context, receptor activation may promote proliferation or apoptosis^81,82^.

The TNFR1 receptor showed reduced levels following treatment with piplartine, and TNFR2 showed no changes in expression. This result indicates that a decreased TNFR1 / TNFR2 ratio compared with the control might alter the cell fate, but no definitive conclusions can be drawn at the moment. These antagonistic effects demonstrate the complexity of the inflammatory process and show that the development of efficient control mechanisms is still a great challenge.

In conclusion, we can assume that PL and other anti-inflammatory compounds show potential interactions with the same peptide sequence of annexin A1. As an anti-inflammatory agent, PL has antiviability effect and is able to regulate the expression of MCP-1 chemokine, IL-8 cytokine and genes that act in several immune signaling pathways and inflammatory diseases. The data confirm an inhibitory effect of PL on tubulin expression, which are in line with the conclusion of Meegan et al.^41^ that piplartine is a tubulin-destabilizing agent. In addition, PL appeared to have no influence on the migration and invasion ability of normal or neoplastic cells, which may be due to a concentration-dependent effect.

The present study confirms the anti-tumor property of piplartine, independently of its interactions with Ac_2-26_, and provides new data on the role of this compound in modulating gene expression, especially of genes related to the inflammatory process. In addition, the study shows that piplartine has physicochemical properties similar to other anti-inflammatory compounds and is a natural immune modulator with potential application in cancer prevention and therapy.

## Materials and methods

### Drugs

The annexin A1-derived peptide Ac_2-26_ (Ac-AMVSEFLKQAWFIENEEQEYVQTVK)^83^ was obtained from THERMO SCIENTIFIC (Waltham, MA, USA) and dissolved in dimethyl sulfoxide (DMSO) at a final concentration in culture medium of 1uM. Piplartine (C_17_H_19_NO_5_; CAS number 20069-09-4) was isolated as previously described^36^ and dissolved in DMSO at a final concentration in culture medium of 10 or 20 µM. PL was also dissolved in absolute ethyl alcohol for UV–Vis absorbance and fluorescence spectroscopy assays. Ac_2-26_ and PL concentrations were determined by experiments previously performed by our group^20,84^ and by other authors^85–87^. Bacterial lipopolysaccharide (LPS, *Escherichia coli* O127:B8, SIGMA ALDRICH, Poole, Dorset, UK), a component of the surface membrane of most gram-negative bacteria and potent stimulator of immune cells, was diluted in 10% MEM-Earle medium (CULTILAB, Campinas, SP, Brazil) at a final concentration in culture medium of 10 µg/mL.

### Molecular docking

Human proteins related to inflammatory and neoplastic processes were selected by literature mining in the PubMed library using the medical subject heading (MeSH) terms of interest. Fourteen proteins with defined three-dimensional structures in the Protein Data Bank were selected for molecular docking studies, including annexin A1. Piplartine and eight anti-inflammatory drugs in current clinical use that meet the Lipinski rules^88^ were used as ligands. Their structures were obtained from the Zinc Database.

Proteins were prepared using AUTODOCK tools (version 1.4.5). Water molecules were deleted, and the hydrogen atoms and Gasteiger charges were added to the receptor molecule. Docking of ligands to protein was performed using AUTODOCK VINA (version 1.1.2 for LINUX)^89^. The grid box was defined to include the binding site of the proteins, and the ligands were docked sequentially. PYMOL software was used to display the proteins with the ligand binding site.

### UV–Vis absorbance and fluorescence spectroscopy

UV–Vis absorption and fluorescence spectroscopic studies were performed to investigate ligand-annexin A1 peptide interactions. Spectra were recorded at room temperature between 200 and 500 nm (integration time of 0.333 s) on a CARY 3E UV–Vis spectrophotometer (VARIAN, Palo Alto, CA, USA) equipped with tungsten and deuterium lamps and using quartz cuvettes with an optical pathlength of 10 mm.

Fluorometric titrations were carried out in 10 mm quartz cuvettes on a PC1 spectrofluorometer (ISS, Champaign, IL, USA) with a NESLAB RTE-221 thermostat bath, running on VINCI software. Excitation wavelengths were fixed at 280 and 295 nm to excite phenylalanine and tryptophan residues, respectively. The excitation and emission slit widths were set to 8 nm. The emission spectra were in the range of 295 to 500 nm with a 1.0 nm resolution step, and spectra were obtained by averaging 10 successive accumulations. Aliquots of piplartine were titrated in 5.0 μM annexin A1-derived peptide solution. The measurements were performed at temperatures of 288, 298 and 308 K. The piplartine concentration varied from 0 to ~ 15.6 μM with increments of 1.2 μM at 288, 298 and 308 K. In all experiments, the final volume of ethyl alcohol in the buffer was ≤ 0.6%, and the fluorescent signal intensity was corrected for the background fluorescence and inner filter effects^90^.

### Cell culture and drug treatments

Two cell lines derived from normal or neoplastic tissues were used in the present study, the HUVEC cell line (CRL-2873; AMERICAN TYPE CULTURE COLLECTION/ATCC, Manassas, VA, USA), which was obtained from normal human umbilical vein/vascular endothelium, and the HEp-2 cell line (CCL-23; ATCC), which was originally established from an epidermoid carcinoma of the larynx. HUVEC was selected for the present study because it is one of the most popular cell lines used for many studies, including for experiments on inflammation^91^. Hep-2 cells were used as a cancer cell model with similar epithelial origin of HUVEC cells^92^.

HUVEC and HEp-2 cells were cultured in MEM-Earle medium (CULTILAB) supplemented with 10% FBS, 10 mM nonessential amino acids (GIBCO, Carlsbad, CA, USA), 2 mM l-glutamine, 1 mM sodium pyruvate (SIGMA ALDRICH) and 0.1% antibiotic/antimycotic (SIGMA ALDRICH), in a humidified atmosphere with 5% CO_2_ at 37 °C.

To investigate whether piplartine in the presence of annexin A1 plays a role in inflammatory and neoplastic processes, HUVECs and HEp-2 cells were treated with the peptide Ac_2-26_, piplartine, both Ac_2-26_ and piplartine, or LPS.

### Cell proliferation and viability assays

The proliferation and viability of HUVEC and HEp-2 cell lines was assessed using the CELLTITER 96 AQUEOUS ONE SOLUTION CELL PROLIFERATION ASSAY/MTS (PROMEGA, Madison, MI, USA). Cells were seeded in 96-well plates at a density of 2 × 10^3^ cells/well in 200 µL complete medium, according to the manufacturer’s instructions. The peptide Ac_2-26_ and PL were dissolved in DMSO and added to cell cultures (1 μl/well) at final concentrations of 1uM Ac_2-26_, 10 µM PL. An equivalent volume of vehicle DMSO without PL or Ac_2-26_ was added to the DMSO control group, and no DMSO/PL/Ac_2-26_ to the negative control. Twenty µL CELLTITER 96 AQUEOUS ONE SOLUTION were then added to each well, and the cells were incubated for 1 h at 37 °C in a humidified atmosphere with 5% CO_2_ and analyzed on a TP-READER NM (THERMOPLATE, EQUIPAR, Curitiba, PR, Brazil) at 490 nm to establish baseline readings. Successive readings were performed every 24 h from plating to 48 h and 72 h after seeding to establish viability curves. Assays were carried out in triplicate, and experiments were performed two times.

### Cell migration and invasion assays

Quantitative evaluation of in vitro migration and invasion assays was performed using HUVEC and HEp-2 cells and a BD BIOCOAT MIGRATION/INVASION CHAMBERS (BD BIOSCIENCES, San Jose, USA). The cells (5 × 10^4^ suspended in 300 µL serum-free MEM) were seeded in the upper compartment of an 8-μm Boyden chamber and treated with PL alone or in combination with peptide Ac_2-26_, (both dissolved in DMSO), at final concentrations of 1uM Ac_2-26_ and 10 µM PL. An equivalent volume of vehicle DMSO without PL or Ac_2-26_ was added to the DMSO control group, and no DMSO/PL/Ac_2-26_ to the negative control. Migration and invasion assays were carried out with uncoated or coated polycarbonate filters, respectively. The bottom chamber was filled with 500 µL medium with 10% FBS. After incubation for 24 h (migration) or 48 h (invasion), the nonmigrated cells on the upper surface of the filter were carefully removed. Cells that migrated to the lower surface of the insert were fixed with 0.4% formaldehyde for 20 min and stained with 4′,6-diamidino-2-phenylindole (DAPI, UNISCIENCE, São Paulo, SP, Brazil). Images of five fields were captured with an inverted fluorescence microscope using AXIOVISION software (Release 4.8, CARL ZEISS, Oberkochen, Germany). Cells were quantified by IMAGEJ software*.* Assays were carried out in triplicate.

### Real-time PCR

Total RNA from HEp-2 cells treated with PL (20 μM) or vehicle control was extracted using TRIzol (LIFE TECHNOLOGIES, Grand Island, NY, USA), and the concentration was determined by a NANODROP ND-1000 (THERMO SCIENTIFIC). RNA was reverse transcribed into complementary DNA (cDNA) using a HIGH CAPACITY CDNA REVERSE TRANSCRIPTION KIT (APPLIED BIOSYSTEMS, Foster City, CA, USA). A PCR array (TAQMAN ARRAY HUMAN INFLAMMATION 96-WELL PLATE, Fast, THERMO FISHER SCIENTIFIC) was screened according to the manufacturer's instructions on an ABI PRISM 7500 FAST REAL-SEQUENCE DETECTION SYSTEM (APPLIED BIOSYSTEMs). The results were analyzed using Data PCR array in the DATAASSIST software (version 3.01, THERMO FISHER SCIENTIFIC). Differentially expressed genes were imported into DAVID^49,50^, a database for annotation, visualization and integrated discovery and Benjamini–Hochberg method was used to control the false discovery rate (FDR)^93^. The genes were annotated for gene ontology and pathways using the whole human genome as a background. INGENUITY PATHWAY ANALYSIS (IPA) software (QIAGEN, Redwood City, CA, USA) was also used to identify relevant canonical pathways, diseases and biological functions overrepresented in differentially expressed genes^51^.

PCR array results were analyzed using the ∆∆Ct method (PCR ARRAY DATA ANALYSIS: https://www.thermofisher.com/order/catalog/product/4418719#/4418719, THERMO FISHER). The array contained 3 housekeeping genes (*GAPDH*, *HPRT1*, *GUSB*) that were used for normalization of the data. The relative expression level of genes involved in inflammatory processes to endogenous control genes was calculated as 2-ΔCT, where ΔCT = CT (gene involved in inflammatory processes) − CT(housekeeping genes). To determine fold change in gene expression, the normalized expression of each gene (case group) was divided by the normalized expression of the same gene (control group), also called 2-ΔΔCT, where ΔΔCT = CT(case group) − CT(control group). Genes with log2 fold change > 1.0 were considered as differentially expressed.

### Western blotting assays

HUVEC cells from the chemokine and cytokine experiments were lysed by syringe passage in ice-cold RIPA buffer (1% NP-40, 0.25% sodium deoxycholate, 150 mM NaCl, 1 mM EDTA, 1 mM PMSF, 1 mM sodium orthovanadate, and 50 mM Tris–HCl at pH 7.4) supplemented with a protease inhibitor cocktail (SIGMA ALDRICH). Proteins from the cell lysates were separated by electrophoresis using a 15% SDS-PAGE polyacrylamide gel and then transferred to a nitrocellulose membrane (MILLIPORE, Bedford, MA, USA). The blotted membrane was stained with a 10% solution of Ponceau red (SIGMA ALDRICH) and blocked with 3% TBS-T-milk (140 mM NaCl, 20 mM Tris–HCl pH 7.4, 0.1% Tween-20, 3% milk powder) for 1 h and washed three times with TBS buffer. The part of the blot, containing proteins larger than 46 kDA, was incubated with rabbit anti-α-tubulin (1/5000, SIGMA ALDRICH) and the part of the blot containing proteins below 46 kDa was incubated with rabbit anti-β-actin mAb (1/5000, CELL SIGNALING TECHNOLOGY, Danvers, MA) in 3% TBS-T milk overnight at 4 °C. The membranes were then washed three times with TBS buffer and incubated with secondary anti-rabbit and anti-mouse antibodies (1:1000, JACKSON IMMUNO RESEARCH, West Grove, PA, USA) in 3% TBS-T-milk for 1 h and washed three times with TBS-T buffer (protocol adapted from). Both parts of the blot were visualized and developed with the enhanced chemiluminescence (ECL) method at the same time. Levels of β-actin were used as endogenous controls. The signal intensity of α-tubulin and β-actin was analyzed using IMAGEJ analysis software^94^. Fold change relative to control was calculated using α-tubulin: β-actin ratio.

### MCP-1 chemokine and IL-1β and IL-8 cytokines

For chemokine and cytokine experiments, HUVEC cultures were treated for 24 and 72 h with LPS, PL and peptide Ac_2- 26_ at final concentrations of 10 μg/mL, 1uM and 10 µM, respectively. No LPS/PL/Ac_2-26_ was added to the negative control. The MCP-1 chemokine and IL-8 and IL-1β cytokines in the supernatant of the culture medium were quantified using ELISA immunoassays (BD BIOSCIENCES, San Diego, CA, USA). Cytokine concentrations from three independent assays were determined by a microplate reader (TP-READER NM THERMOPLATE, EQUIPAR) according to the manufacturer's instructions. Concentrations were expressed as the mean ± standard error of the mean (SEM) of cytokine concentrations (pg/mL).

### Statistical analysis

Analysis of variance (ANOVA) was applied followed by the Bonferroni test with *p* < 0.05. The results are shown as the mean ± standard error of the mean (SEM).

## Supplementary information

Supplementary Information.
