# Supplementary material for: Biological and physical approaches on the role of piplartine (piperlongumine) in cancer
Source: Sci Rep. 2020 Dec 17;10:22283. doi: 10.1038/s41598-020-78220-6 (PMC7746756; doi:10.1038/s41598-020-78220-6)
Supplement: Supplementary file 1 — Supplementary Information. [file 41598_2020_78220_MOESM1_ESM.pdf]

SUPPLEMENTARY INFORMATION FILE

Supplementary Material

Biological and physical approaches on the role of piplartine (piperlongumine) in cancer

Tiago Henrique[1], Caroline de F. Zanon[2], Ana P. Girol[2,3], Ana Carolina Buzzo Stefanini[1,9], Nayara S. de A. Contessoto[4], Nelson J. F. da Silveira[5], Daniel P. Bezerra[6], Edilberto R. Silveira[7], José M. Barbosa-Filho[8], Marinonio L. Cornélio[4], Sonia M. Oliani[2] and Eloiza H. Tajara[1,9*]

[1]Department of Molecular Biology, School of Medicine of São José do Rio Preto (FAMERP), São José do Rio Preto, SP, 15090-000, Brazil.

[2]Department of Biology, São Paulo State University (UNESP), Institute of Biosciences, Humanities and Exact Sciences (IBILCE), Campus São José do Rio Preto, SP, 15054-000, Brazil.

[3]Integrated College Padre Albino Foundation (FIPA), Catanduva, SP, 15806-310, Brazil.

[4]Departament of Physics, São Paulo State University (UNESP), Institute of Biosciences, Humanities and Exact Sciences (IBILCE), Campus São José do Rio Preto, SP, 15054-000, Brazil.

[5]Institute of Exact Science, Federal University of Alfenas, Alfenas, MG, 37130-000, Brazil.

[6]Gonçalo Moniz Institute, Oswaldo Cruz Foundation (IGM-FIOCRUZ/BA), Salvador, BA, 40296-710, Brazil.

[7]Department of Chemistry, Federal University of Ceará, Fortaleza, CE, 60020-181, Brazil.

[8]Laboratory of Pharmaceutics Technology, Federal University of Paraiba, João Pessoa, PB, 58051-085, Brazil.

[9]Department of Genetics and Evolutive Biology, Institute of Biosciences, University of São Paulo, São Paulo, SP, 05508-090, Brazil.

TH and CFZ contributed equally to this work.

*Correspondence should be addressed to: Eloiza Helena Tajara, PhD, Department of Molecular Biology, School of Medicine/FAMERP, São José do Rio Preto, Av Brig Faria Lima 5416, CEP 15090-000, São José do Rio Preto, SP, Brazil. Phone: +55 17 3201-5809, e-mail: tajara@famerp.br

Physicochemical Analysis details

Molecular Docking

Supplementary Table S1 presents Lipinski parameters for PL and eight common anti-inflammatory compounds in current clinical use. **Supplementary Table S2** presents the values of free energy obtained by docking analysis of 14 human proteins related to inflammatory and neoplastic processes with defined three-dimensional structures as targets, and PL and eight anti-inflammatory drugs as ligands. All RMSD (Root Mean Square Deviation) values were equal to zero. The results indicate that the score obtained is sufficient for successful identification of protein-ligand interactions. However, definitive confirmation of these results requires crystallography experiments, which limit the risk of false positives.

Supplementary Figure S1a shows the docked position of PL and eight anti-inflammatory compounds onto N-terminal sequence of ANXA1 corresponding to the Ac2-26 peptide.

Homology Modeling

A homology model of the peptide Ac_{2-26} was built using the SWISS-MODEL server [46]. A high similarity between the predicted peptide sequence and the full-length ANXA1 was observed (**Supplementary Figure S1b, c).**

Physicochemical Analysis

Physicochemical properties of the peptide Ac_{2-26} and PL interaction were obtained by fluorescence spectroscopy analysis. The emission spectra were analyzed by the addition of PL into annexin A1-derived peptide solution (**Supplementary Fig. S2**) at excitation wavelength of 295 nm and temperatures of 288, 298 and 308 K.

Supplementary Table S3 presents excitation wavelength (λ_{ex}), temperature (T) in Kelvin, Stern-Volmer quenching constant (K_{SV}), affinity constant (K_a), stoichiometric number (n), and thermodynamic parameters [Gibbs free energy (ΔG^o), enthalpy (ΔH^o), entropy (ΔS^o)] of the peptide Ac_{2-26} and PL interaction at pH 7.0 and temperature 288, 298 and 308 K.

The quencher mechanism was determined using the Stern-Volmer analysis and the **Equation (1)** [47].

$$\frac{Fo}{F} = 1 + K_{SV}[PIP]$$ **Equation (1)**

F_o and F are the fluorescence intensities of peptide Ac2-26 in the absence and presence of PL, respectively. K_{sv} is the Stern-Volmer constant, and [PIP] is the concentration of PL. The results indicated an increase of the Stern-Volmer constant when the temperature increases, suggesting a dynamic quencher mechanism (**Supplementary Fig. S3** and **Supplementary Table S3**).

The affinity constants (K_a) and stoichiometric numbers (n) between the peptide and PL for each temperature and excitation wavelength of 295 nm were determined using the double-logarithmic analysis - **Equation (2)** [48].

$$log\ \frac{Fo-F}{F} = logK_a + n\ log\ [PIP]$$ **Equation (2)**

Gene and Protein Expression Analysis details

PL Inhibits α-Tubulin Expression

The original, unprocessed images of the blots shown in **Fig. 5** (main text of the manuscript) are presented in **Supplementary Fig S4**.

PL Modulates Gene Expression

The **Supplementary Table S4** shows the molecular functions annotated for differentially expressed genes using DAVID database [49,50].

REFERENCES ARE LISTED IN THE MAIN TEXT

a

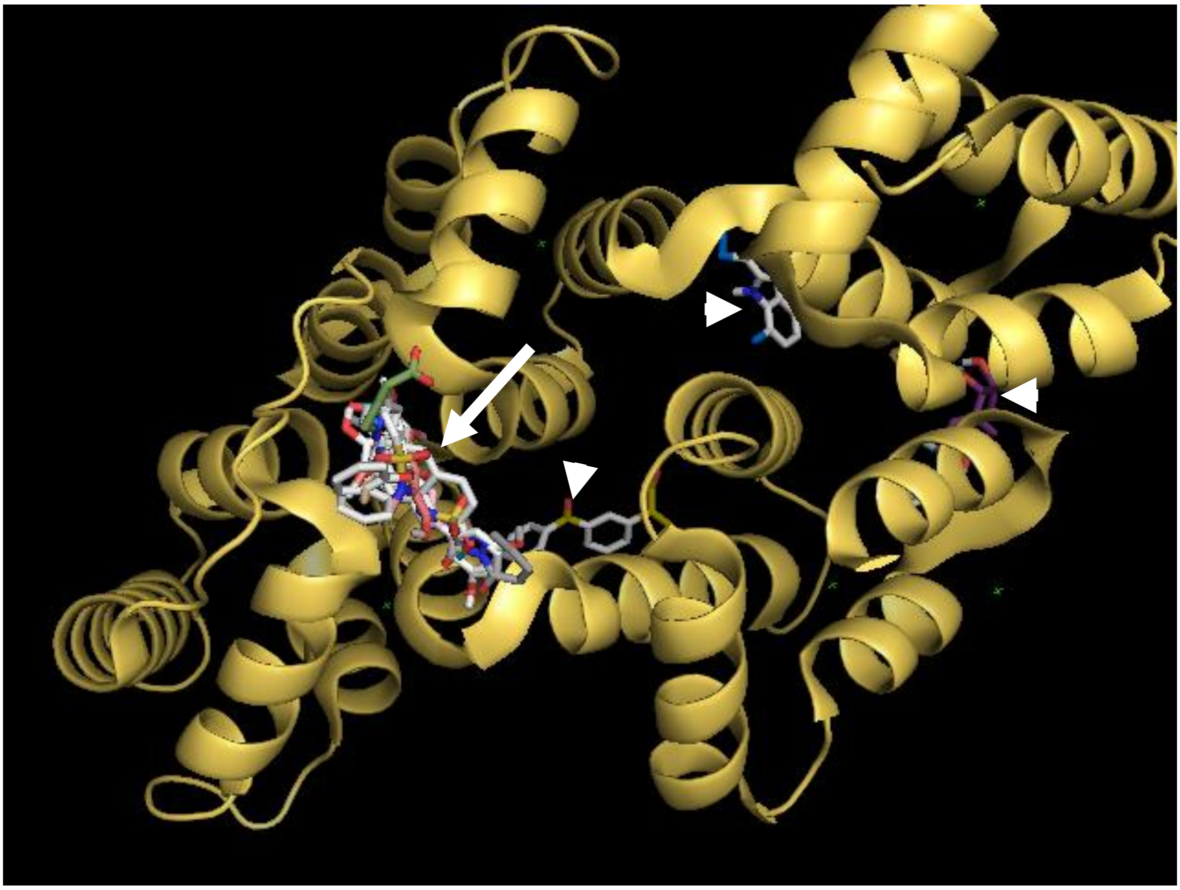

b **c**

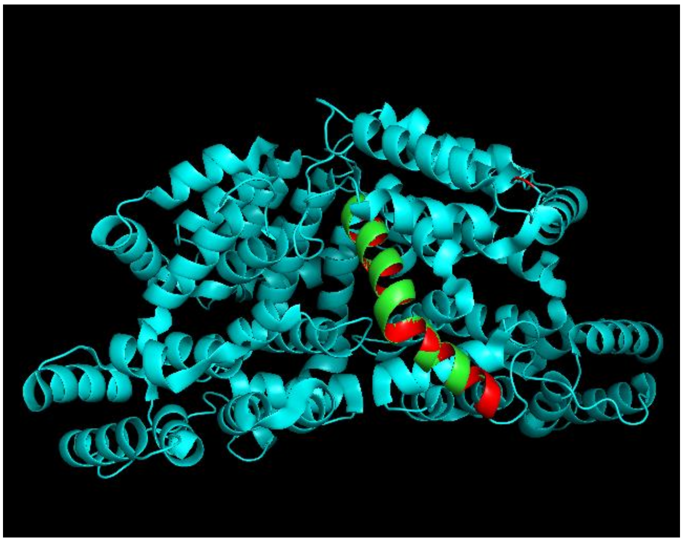

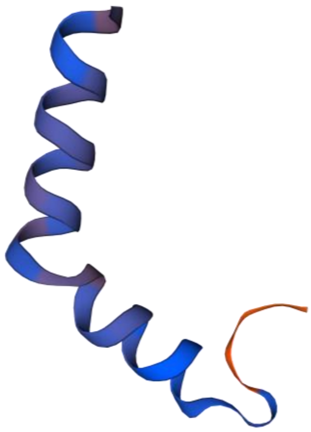

Supplementary Figure S1. (**a**) **The docked position of PL and eight anti-inflammatory drugs in current clinical use onto $Ac_{2\text{-}26}$.** The secondary structure of annexin A1 is represented in yellow, and drugs and PL are shown as sticks. Arrows= acetaminophen, ketorolac, naproxen, nimesulide, resveratrol, and PL; arrowheads= dexamethasone, diclofenac sodium, and ketoprofen. Data were obtained, with permission, from the thesis of Henrique T [95], and figure printed in [96]. (**b**) The superposition of the peptide $Ac_{2\text{-}26}$ model (highlighted in red) on the N-terminal sequence of ANXA1 (in green). (**c**) isolated peptide $Ac_{2\text{-}26}$ model. Figures (a) and (b) were generated by Pymol system version 2.0 (https://pymol.org/). Figure (c) was built by the SWISS-MODEL online [46] (https://swissmodel.expasy.org/).

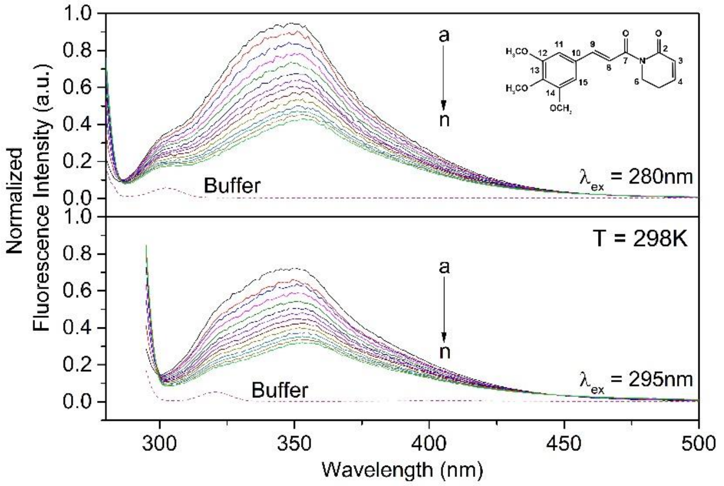

Supplementary Figure S2. Emission spectra of annexin A1-derived peptide $Ac_{2\text{-}26}$ in absence or presence of piplartine. Emission spectra of the peptide (5.0 μM) in the absence (a) and presence of PL (b-n: 1.2-15.6 μM) at 280 nm and 295 nm excitation wavelengths. The arrow indicates the progress of titration, with increments of 1.2 μM (pH 7.0, T = 298 K). The dashed line represents the emission spectrum of the phosphate buffer. Insert: chemical structure of PL. Data were taken, with permission, from experiments performed by the authors Contessoto NSA. and Cornélio ML, and figure printed in [96].

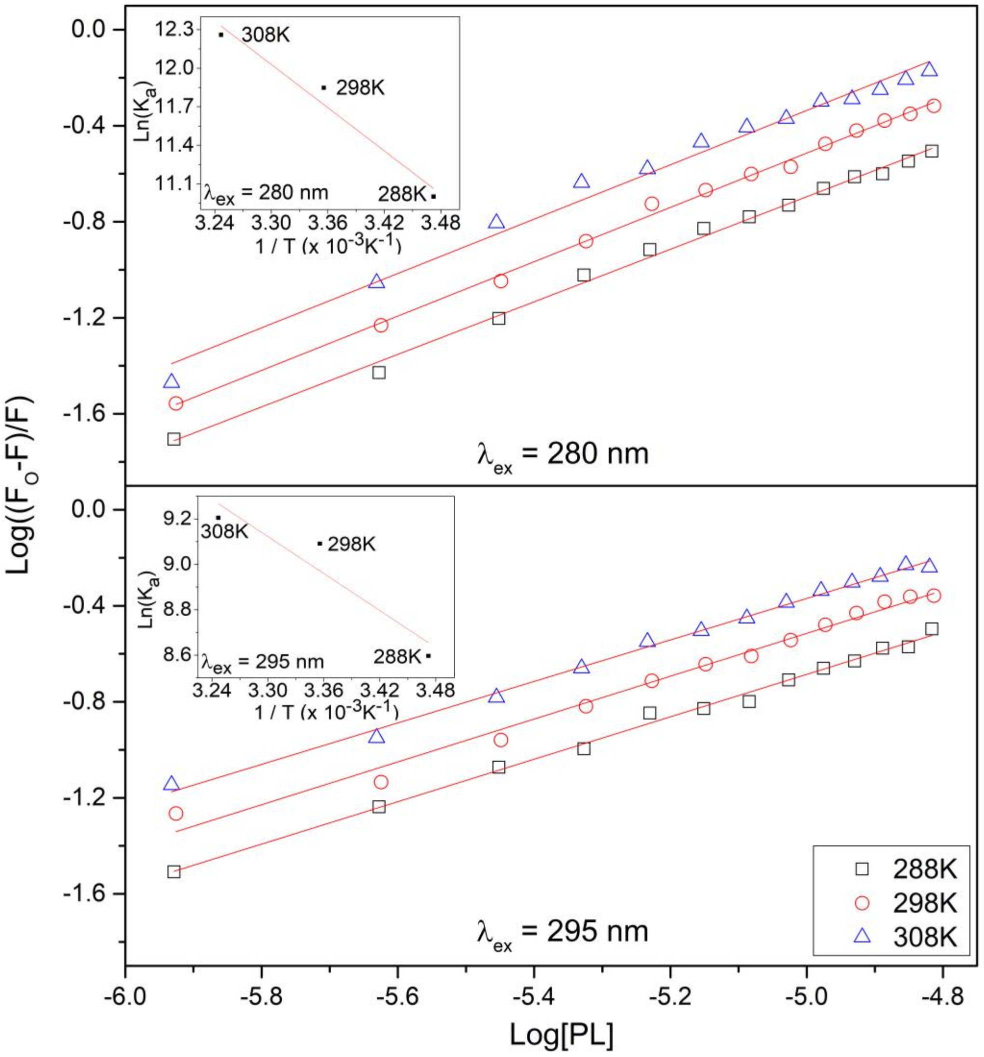

Supplementary Figure S3. Double-log plots using fluorescence excitation (λ_{ex}) wavelengths at 280 and 295nm for the peptide Ac_{2-26} by titration of PL with temperature fixed at 288, 298 and 308K. The inserts show the Van't Hoff plots for each excitation wavelength. Data were taken, with permission, from experiments performed by the authors Contessoto NSA and Cornélio ML, and figure printed in [96].

a

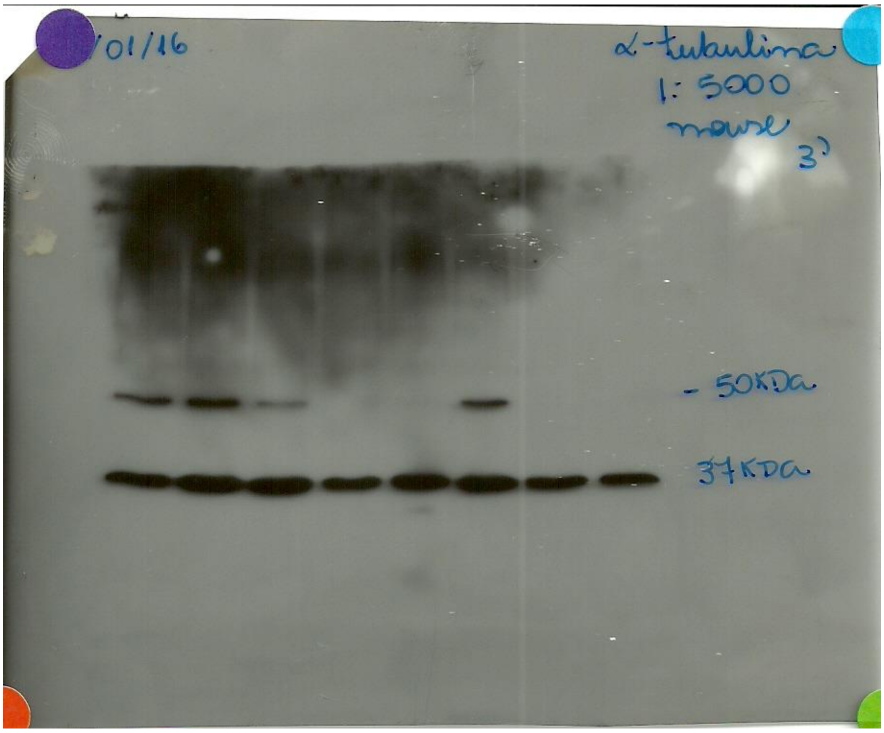

b

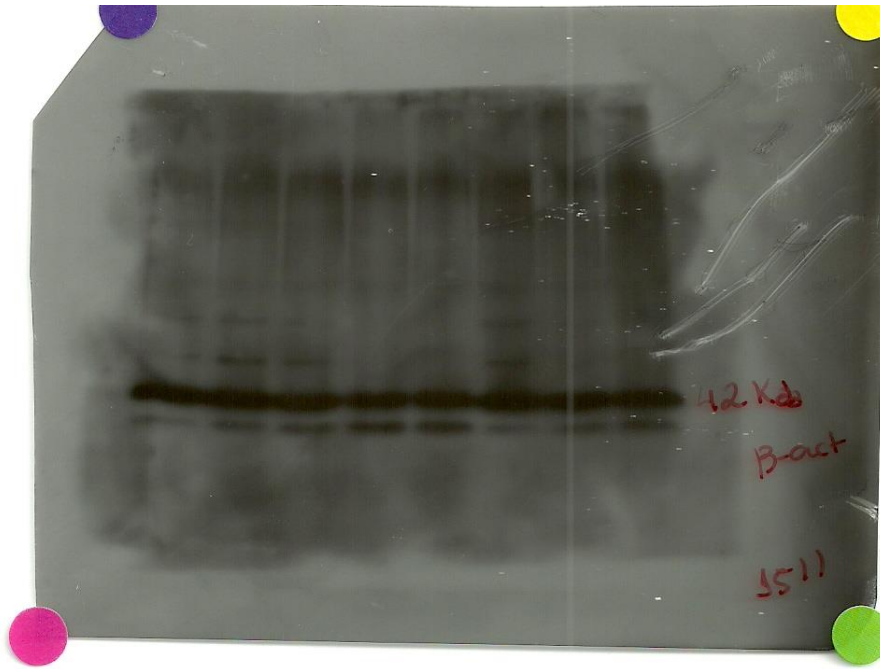

Supplementary Figure S4. Original, unprocessed images of the blots shown in Fig. 5. (**a**) 50kDa band: α-tubulin; 37kDa band: annexin A1; (**b**) 42kDa band: β-actin.

Supplementary Table S1. Lipinski parameters for PL and eight common anti-inflammatory compounds in current clinical use. Reproduced with permission from [95].

Ligand	Zinc code	Molecular weight (g/mol)	Log P	Number of H-bond donors	Number of H-bond acceptors
Acetaminophen	18274777	151.165	0.68	2	3
Dexamethasone	03875332	392.467	2.06	3	5
Diclofenac sodium	00001281	295.145	4.57	1	3
Ketoprofen	00002272	253.277	3.59	0	3
Ketorolac	00002279	254.265	2.20	0	4
Naproxen	00105216	229.255	3.38	0	3
Nimesulide	04617749	307.307	2.81	0	7
Resveratrol	00006787	228.247	0.79	3	3
PL	00899053	317.341	1.62	0	6

Supplementary Table S2. Docking analysis: PL exhibits physicochemical properties similar to those of the anti-inflammatory drugs. PDB identification (ID) and binding free energy values obtained by docking analysis of 14 proteins related to inflammatory and neoplastic processes as targets, and PL and eight anti-inflammatory drugs as ligands. All RMSD (Root Mean Square Deviation) were equal to zero.

Target	PDB ID	Ligand	Free energy	Target	PDB ID	Ligand	Free energy
**ANXA1**	1MCX	Acetaminophen	-4.7 kcal/mol	**MAPK1**	4O6E	Acetaminophen	-5.2 kcal/mol
		Dexamethasone	-6.8 kcal/mol			Dexamethasone	-8.2 kcal/mol
		Diclofenac	-6.6 kcal/mol			Diclofenac	-6.5 kcal/mol
		Ketoprofen	-6.1 kcal/mol			Ketoprofen	-7.2 kcal/mol
		Ketorolac	-6.2 kcal/mol			Ketorolac	-7.1 kcal/mol
		Naproxen	-5.9 kcal/mol			Naproxen	-7.4 kcal/mol
		Nimesulide	-5.6 kcal/mol			Nimesulide	-6.4 kcal/mol
		PL	-6.8 kcal/mol			PL	-6.8 kcal/mol
		Resveratrol	-6.7 kcal/mol			Resveratrol	-6.8 kcal/mol
**CD40**	3LKJ	Acetaminophen	-6.3 kcal/mol	**MAPK3**	4QTB	Acetaminophen	-6.0 kcal/mol
		Dexamethasone	-7.8 kcal/mol			Dexamethasone	-9.5 kcal/mol
		Diclofenac	-7.8 kcal/mol			Diclofenac	-7.5 kcal/mol
		Ketoprofen	-9.0 kcal/mol			Ketoprofen	-9.2 kcal/mol
		Ketorolac	-8.8 kcal/mol			Ketorolac	-8.9 kcal/mol
		Naproxen	-8.6 kcal/mol			Naproxen	-8.6 kcal/mol
		Nimesulide	-8.6 kcal/mol			Nimesulide	-8.0 kcal/mol
		PL	-8.9 kcal/mol			PL	-8.1 kcal/mol
		Resveratrol	-8.4 kcal/mol			Resveratrol	-8.7 kcal/mol
**CD40LG**	1ALY	Acetaminophen	-4.1 kcal/mol	**MAPK8**	3ELJ	Acetaminophen	-6.1 kcal/mol
		Dexamethasone	-6.1 kcal/mol			Dexamethasone	-7.6 kcal/mol
		Diclofenac	-5.0 kcal/mol			Diclofenac	-7.5 kcal/mol
		Ketoprofen	-6.0 kcal/mol			Ketoprofen	-8.5 kcal/mol
		Ketorolac	-5.7 kcal/mol			Ketorolac	-7.8 kcal/mol
		Naproxen	-5.5 kcal/mol			Naproxen	-8.0 kcal/mol
		Nimesulide	-5.2 kcal/mol			Nimesulide	-8.0 kcal/mol
		PL	-5.5 kcal/mol			PL	-8.1 kcal/mol
		Resveratrol	-5.6 kcal/mol			Resveratrol	-7.5 kcal/mol
**HPGD**	2GDZ	Acetaminophen	-6.2 kcal/mol	**MAPK14**	5LAR	Acetaminophen	-6.3 kcal/mol
		Dexamethasone	-7.9 kcal/mol			Dexamethasone	-8.0 kcal/mol
		Diclofenac	-6.9 kcal/mol			Diclofenac	-7.8 kcal/mol
		Ketoprofen	-8.4 kcal/mol			Ketoprofen	-9.6 kcal/mol
		Ketorolac	-8.3 kcal/mol			Ketorolac	-9.3 kcal/mol
		Naproxen	-8.3 kcal/mol			Naproxen	-9.0 kcal/mol
		Nimesulide	-8.1 kcal/mol			Nimesulide	-8.1 kcal/mol
		PL	-8.2 kcal/mol			PL	-7.6 kcal/mol
		Resveratrol	-8.8 kcal/mol			Resveratrol	-8.9 kcal/mol
**IL13**	5L6Y	Acetaminophen	-5.2 kcal/mol	**NOS2**	3EJ8	Acetaminophen	-4.5 kcal/mol
		Dexamethasone	-8.2 kcal/mol			Dexamethasone	-7.2 kcal/mol
		Diclofenac	-5.7 kcal/mol			Diclofenac	-6.0 kcal/mol
		Ketoprofen	-6.9 kcal/mol			Ketoprofen	-6.4 kcal/mol
		Ketorolac	-6.9 kcal/mol			Ketorolac	-6.1 kcal/mol
		Naproxen	-6.1 kcal/mol			Naproxen	-6.2 kcal/mol
		Nimesulide	-6.2 kcal/mol			Nimesulide	-5.9 kcal/mol
		PL	-6.6 kcal/mol			PL	-6.0 kcal/mol
		Resveratrol	-6.3 kcal/mol			Resveratrol	-6.0 kcal/mol
**IL1R1**	1ITB	Acetaminophen	-6.2 kcal/mol	**PTGIS**	3B6H	Acetaminophen	-5.7 kcal/mol
		Dexamethasone	-8.3 kcal/mol			Dexamethasone	-8.7 kcal/mol
		Diclofenac	-6.6 kcal/mol			Diclofenac	-6.7 kcal/mol
		Ketoprofen	-7.3 kcal/mol			Ketoprofen	-7.8 kcal/mol
		Ketorolac	-7.5 kcal/mol			Ketorolac	-7.6 kcal/mol
		Naproxen	-7.5 kcal/mol			Naproxen	-6.8 kcal/mol
		Nimesulide	-6.7 kcal/mol			Nimesulide	-7.1 kcal/mol
		PL	-7.3 kcal/mol			PL	-7.5 kcal/mol
		Resveratrol	-6.9 kcal/mol			Resveratrol	-7.4 kcal/mol
**IL2RA**	2B5I	Acetaminophen	-5.6 kcal/mol	**TNF**	2AZ5	Acetaminophen	-5.7 kcal/mol
		Dexamethasone	-7.1 kcal/mol			Dexamethasone	-8.5 kcal/mol
		Diclofenac	-6.6 kcal/mol			Diclofenac	-7.1 kcal/mol
		Ketoprofen	-7.4 kcal/mol			Ketoprofen	-7.8 kcal/mol
		Ketorolac	-7.1 kcal/mol			Ketorolac	-7.4 kcal/mol
		Naproxen	-6.5 kcal/mol			Naproxen	-7.4 kcal/mol
		Nimesulide	-6.7 kcal/mol			Nimesulide	-6.8 kcal/mol
		PL	-7.0 kcal/mol			PL	-7.1 kcal/mol
		Resveratrol	-7.4 kcal/mol			Resveratrol	-7.7 kcal/mol

Supplementary Table S3. **Peptide Ac2-26 and PL interaction.** Excitation wavelength (λ_{ex}), temperature (T) in Kelvin, Stern-Volmer quenching constant (K_{SV}), affinity constant (K_a), stoichiometric number (n), and thermodynamic parameters [Gibbs free energy (ΔG°), enthalpy (ΔH°), entropy (ΔS°)] of the peptide $Ac_{2\text{-}26}$ and PL interaction at pH 7.0 and temperature 288, 298 and 308 K.

λ_{ex} (nm)	T (K)	K_{SV} ($x10^4$ M^{-1})	K_a ($x10^4$ M^{-1})	n	ΔG° (kJ mol^{-1})	ΔH° (kJ mol^{-1})	ΔS° (kJ mol^{-1})
280	288	2.05	5.992	1.09	-26.328	46.541	72.869
	298	3.24	13.974	1.13	-29.339		75.880
	308	4.51	21.089	1.13	-31.377		77.918
295	288	1.95	0.542	0.88	-20.575	22.557	43.132
	298	2.97	0.887	0.89	-22.513		45.070
	308	3.94	0.995	0.87	-23.560		46.117

Correlation coefficient ≥ 0.794.

Table S4: Molecular functions annotated for gene ontology using DAVID database. Differentially expressed genes identified by a PCR array (TaqMan® Array Human Inflammation 96-well plate) in HEp-2 cells treated with PL compared to untreated cells.

Category	Term	Count	PValue	Genes	List Total	Bonferroni	Benjamini	FDR
GOTERM_MF_DIRECT	GO:0004629~phospholipase C activity	4	4.025420086822375E-6	PLCB3, PLCE1, PLCG1, PLCG2	43	6.72020600477774E-4	6.72020600477774E-4	0.00489607058322683
GOTERM_MF_DIRECT	GO:0004974~leukotriene receptor activity	3	3.6144360554455966E-5	CYSLTR1, LTB4R, LTB4R2	43	0.006018035941809052	0.0030135587390413	0.0439540755754364
GOTERM_MF_DIRECT	GO:0004435~phosphatidylinositol phospholipase C activity	4	4.018249527869714E-5	PLCB3, PLCE1, PLCG1, PLCG2	43	0.006688145664741962	0.002234370581844966	0.048863629101358885
GOTERM_MF_DIRECT	GO:0005057~receptor signaling protein activity	4	1.3270751575805687E-4	PLCE1, ADRB1, PLCG1, IL1RL1	43	0.021919817199035907	0.005525583942103451	0.16129469141312747
GOTERM_MF_DIRECT	GO:0004707~MAP kinase activity	3	5.395986202349226E-4	MAPK1, MAPK14, MAPK8	43	0.08619428040993349	0.017865935595569926	0.6543490218670067
GOTERM_MF_DIRECT	GO:0008528~G-protein coupled peptide receptor activity	3	9.015294019882727E-4	CYSLTR1, LTB4R, LTB4R2	43	0.13982834947094525	0.024791403756451946	1.0910431835692402
GOTERM_MF_DIRECT	GO:0019899~enzyme binding	6	0.0013547160852595823	A2M, PLCE1, MAPK14, MAPK8, PDE4D, CD40	43	0.2025937201266077	0.031824171266769286	1.6353596164319417
GOTERM_MF_DIRECT	GO:0004871~signal transducer activity	5	0.0016182032026242626	PLCB3, PLCE1, PLCG1, PLCG2, CD40	43	0.23697068106482322	0.03324227015184411	1.950565188872233
GOTERM_MF_DIRECT	GO:0001632~leukotriene B4 receptor activity	2	0.004969965392009579	LTB4R, LTB4R2	43	0.5648452552767138	0.08830556769788056	5.880146078263515
GOTERM_MF_DIRECT	GO:0043120~tumor necrosis factor binding	2	0.004969965392009579	TNFRSF1A, A2M	43	0.5648452552767138	0.08830556769788056	5.880146078263515
GOTERM_MF_DIRECT	GO:0019976~interleukin-2 binding	2	0.007445897701565126	IL2RB, IL2RA	43	0.7129553158241384	0.11733694126263783	8.68950527894523
GOTERM_MF_DIRECT	GO:0004911~interleukin-2 receptor activity	2	0.007445897701565126	IL2RB, IL2RA	43	0.7129553158241384	0.11733694126263783	8.68950527894523
GOTERM_MF_DIRECT	GO:0051380~norepinephrine binding	2	0.009915815481849218	ADRB2, ADRB1	43	0.810659040089754	0.14040286107011424	11.415167889108035
GOTERM_MF_DIRECT	GO:0019966~interleukin-1 binding	2	0.014837664441734478	IL1R2, A2M	43	0.9176236502258279	0.1878238875206899	16.625284470493984
GOTERM_MF_DIRECT	GO:0051379~epinephrine binding	2	0.014837664441734478	ADRB2, ADRB1	43	0.9176236502258279	0.1878238875206899	16.625284470493984
GOTERM_MF_DIRECT	GO:0004908~interleukin-1 receptor activity	2	0.017289624032679966	IL1R2, IL1RL1	43	0.9456667799231604	0.20072295217058944	19.11449653915459
GOTERM_MF_DIRECT	GO:0005515~protein binding	30	0.018993291507232873	IL1R2, A2M, TNF, CYSLTR1, CACNB2, NFKB1, BDKRB1, NR3C1, ITGB1, ITGAM, TNFRSF1A, NOS2, HTR3A, KNG1, IL2RB, KLK3, IL1RL1, PDE4D, CD40, MAPK1, ADRB2, PLCE1, ADRB1, PLCG1, MAPK14, PLCG2, TBXA2R, MAPK8, LTA4H, ALOX12	43	0.959335053780849	0.20446629241887126	20.803685094209513
GOTERM_MF_DIRECT	GO:0047498~calcium-dependent phospholipase A2 activity	2	0.022175684216815154	PLA2G2A, PLA2G5	43	0.9763648114373361	0.22094054133426566	23.872598589918546
GOTERM_MF_DIRECT	GO:0002020~protease binding	3	0.026127358396753866	A2M, TNF, ITGB1	43	0.9879811846758512	0.2414375269103105	27.531367555223785
GOTERM_MF_DIRECT	GO:0005088~Ras guanyl-nucleotide exchange factor activity	3	0.03318366978798705	IL2RB, IL2RA, ADRB1	43	0.9964318408609747	0.28216251968342	33.665946914602515
GOTERM_MF_DIRECT	GO:0004115~3',5'-cyclic-AMP phosphodiesterase activity	2	0.03669200559779155	PDE4B, PDE4D	43	0.9980556140846054	0.29306936276145223	36.535160020505685
GOTERM_MF_DIRECT	GO:0004114~3',5'-cyclic-nucleotide phosphodiesterase activity	2	0.05572025620075663	PDE4B, PDE4D	43	0.999930527571776	0.3958454853177139	50.20959619026559
GOTERM_MF_DIRECT	GO:0005031~tumor necrosis factor-activated receptor activity	2	0.058072833567297914	TNFRSF1A, CD40	43	0.9999541971088349	0.3932012959051795	51.697602755968916
GOTERM_MF_DIRECT	GO:0030552~cAMP binding	2	0.058072833567297914	PDE4B, PDE4D	43	0.9999541971088349	0.3932012959051795	51.697602755968916
GOTERM_MF_DIRECT	GO:0004623~phospholipase A2 activity	2	0.07438141746769948	PLA2G2A, PLA2G5	43	0.999997521694571	0.45917720059648126	60.942177903734176
GOTERM_MF_DIRECT	GO:0005245~voltage-gated calcium channel activity	2	0.09494503915006354	CACNB2, CACNB4	43	0.9999999418275728	0.5310532736983736	70.28123430714388
